# (*E*)-5-(4-Methyl­benzyl­idene)-1-phenyl-4,5,6,7-tetra­hydro-1*H*-indazol-4-one

**DOI:** 10.1107/S2414314622002838

**Published:** 2022-03-29

**Authors:** C. Selva Meenatchi, S. Athimoolam, J. Suresh, R. Vishnu Priya, S. Raja Rubina, S. R. Bhandari

**Affiliations:** a Department of Physics, The Madura College, Madurai 625 011, India; b Department of Physics, University College of Engineering Nagercoil, Anna University, Nagercoil 629 004, Tamilnadu, India; c Department of Organic Chemistry, School of Chemistry, Madurai Kamaraj University, Madurai 625 021, India; d Department of Physics, Bhairahawa M. Campus, Tribhuvan University, Nepal; Sunway University, Malaysia

**Keywords:** crystal structure, Hirshfeld surface, indazol-4-one

## Abstract

In the title compound, C_21_H_18_N_2_O, the non-aromatic six-membered ring adopts a distorted envelope conformation with one of the methyl­ene-C atoms being the flap atom. The dihedral angle between the phenyl and 4-tolyl rings is 75.3 (1)°. The 1,2-diazole ring forms dihedral angles of 41.9 (1) and 65.5 (1)° with the phenyl and 4-tolyl rings, respectively. In the crystal, stabilizing C—H⋯O, C—H⋯π and π–π inter­actions are evident. The calculated Hirshfeld surfaces highlight the prominent role of C—H⋯O inter­actions (8.6%), along with H⋯H (51.7%) and C⋯H/H⋯C (29.2%) surface contacts.

## Abstract 2

The 1,2-diazole ring in the title compound is fused to a non-aromatic six-membered ring and bears an N-bound phenyl ring. In the crystal, weak C—H⋯O, C—H⋯π and π–π inter­actions contribute to the three-dimensional architecture.

## Graphical abstract

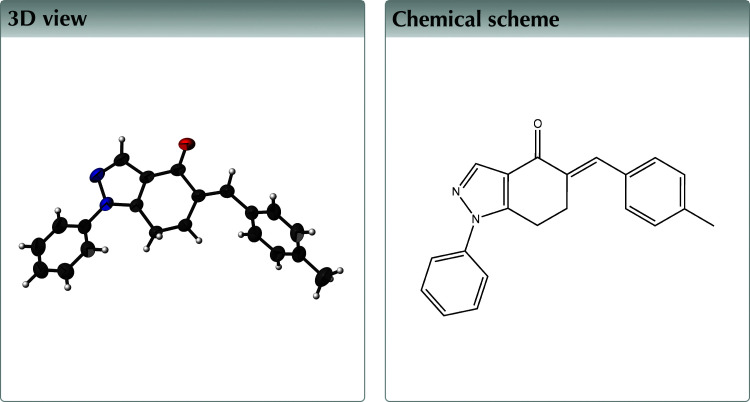

## Structure description

Heterocyclic compounds have been investigated for a long while in view of their pharmaceutical and biological importance. 1,2-Diazole derivatives are found to possess anti-bacterial, anti-viral, anti-inflammatory, anti-depressant and anti-cancer activities (Popat *et al.*, 2003 ▸; Faisal *et al.*, 2019 ▸) because of their conformational freedom and exhibit inter­molecular inter­actions of biological relevance. Owing to its medicinal inter­est and in a continuation of previous work, the crystal and mol­ecular structures of another indazole derivative, namely, (*E*)-5-(4-methyl­benzyl­idene)-1-phenyl-4,5,6,7-tetra­hydro-1*H*-ind­azol-4-one, (I), is reported here.

**Figure 1:**
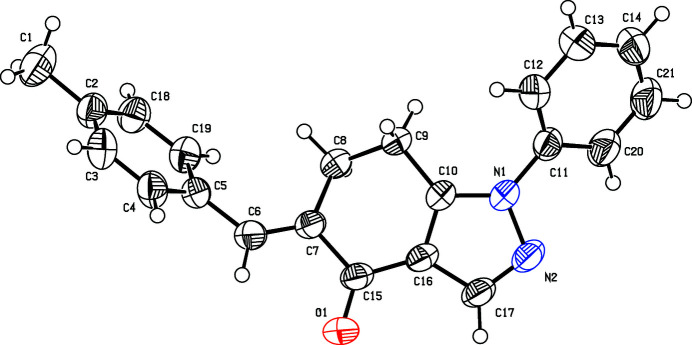
The mol­ecular structure of (I), showing 50% probability displacement ellipsoids

The mol­ecule of (I) and the recently reported 4-chloro­benzyl­idene derivative (II) (Meenatchi *et al.*, 2021 ▸) are isomorphous. The shorter *b*-axis lengths differ slightly between the isomorphous crystal structures, *i.e*. 8.7177 (5) Å for (I) and 8.6604 (5) Å for (II). In (I), the non-aromatic six-membered ring adopts a distorted envelope conformation with the methyl­ene-C9 atom being the flap atom, Fig. 1 ▸. The heterocyclic five-membered ring forms dihedral angles of 41.9 (1) and 65.5 (1)° with the pendent N-bound phenyl and 4-tolyl rings, respectively. A weak intra­molecular C6—H12⋯O1 inter­action (Table 1 ▸) stabilizes the mol­ecular structure.

**Figure 2:**
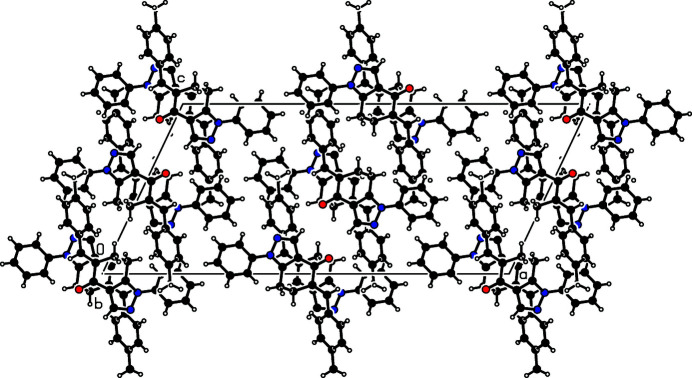
The mol­ecular packing of (I), viewed down the *b* axis.

**Figure 3:**
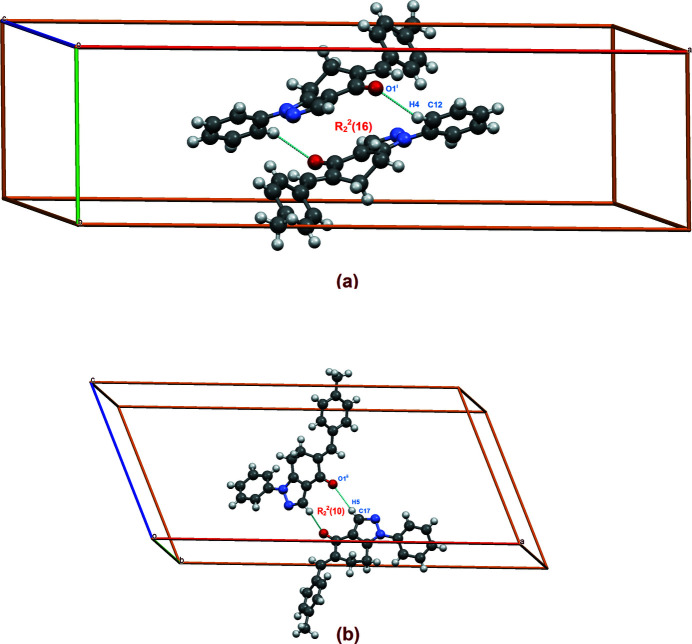
C—H⋯O inter­actions shown as dashed lines forming ring (*a*) 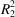 (16) and (*b*) 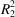 (10) motifs.

**Figure 4:**
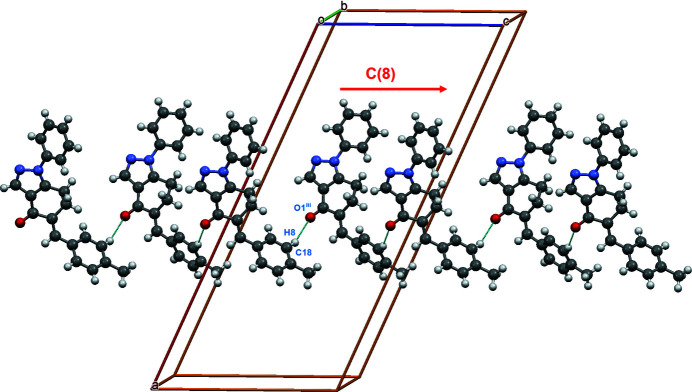
C—H⋯O inter­actions shown as dashed lines forming chain *C*(8) motif along *b* axis of the unit cell

The mol­ecular packing features C—H⋯O, C—H⋯π and π–π inter­actions (Fig. 2 ▸). The C—H⋯O inter­molecular inter­actions, *viz*., C12—H4⋯O1^i^ and C17—H5⋯O1^ii^, lead, respectively, to two centrosymmetric ring 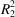 (16) and 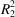 (10) motifs (Bernstein *et al.*, 1995 ▸) (Fig. 3 ▸); see Table 1 ▸ for symmetry operations. These centrosymmetric dimers are connected through another C—H⋯O inter­action, namely, C18—H8⋯O1^iii^, leading to a chain *C*(8) motif along the *c*-axis direction of the unit cell (Fig. 4 ▸).

**Figure 5:**
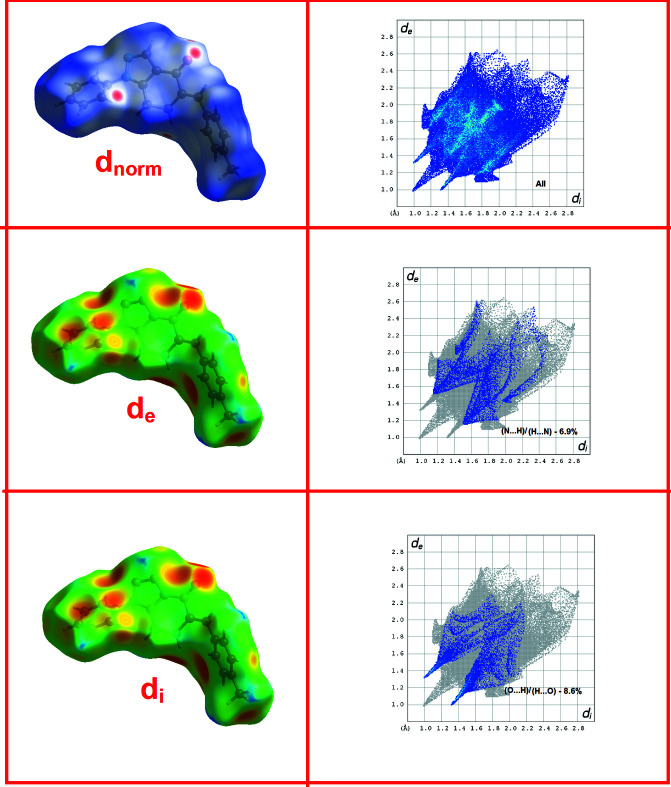
3-D Hirshfeld surfaces (showing *d*_norm_, *d*_i_ and d_e_) and 2-D fingerprint plots.

As a qu­anti­tative approach to analyse the inter­molecular inter­actions, the Hirshfeld surfaces and two-dimensional (2-D) fingerprint plots were generated by employing the *Crystal Explorer* software (Wolff *et al.*, 2012 ▸). The Hirshfeld surface is colour-mapped with the normalized contact distance, *d*_norm_, from red (distances shorter than the sum of the van der Waals radii) through white to blue (distances longer than the sum of the van der Waals radii). The different types of inter­molecular inter­actions can be identified by colour coding the distances from the surface to the nearest atom exterior (*d*_e_) or inter­ior (*d*_i_) plots to the surface. The 2-D fingerprint plots from the surface analysis and the *d*_norm_ surface were analysed for (I) to further explore the packing modes and inter­molecular inter­actions. The 3-D Hirshfeld surfaces and 2-D fingerprint plots with percentage contributions are shown in Fig. 5 ▸. C⋯H/H⋯C contacts (with a pair of spikes in the fingerprint plot, 29.2%) and O⋯H/H⋯O inter­actions (sharp spikes, 8.6%) make a significant contribution to the overall contacts; the latter incorporate the notable C—H⋯O inter­actions. The H⋯H inter­actions contribute 51.7% with widely scattered points of high density showing a large proportion of hydrogen atoms in the mol­ecular structure, indicating the importance of van der Waals contacts in the mol­ecular packing. The N⋯H/H⋯N inter­molecular contacts are identified as making a notable contribution to the total Hirshfeld surface comprising about 6.9%. However, the C—H⋯N inter­molecular inter­actions are not prominent in the packing as the separations are greater than the van der Waals radii.

## Synthesis and crystallization

A mixture of 1-phenyl-1,5,6,7-tetra­hydro-4*H*-indazol-4-one (1 mmol) and 4-methyl­benzaldehyde (1 mmol) was dissolved in ethanol followed by the addition of alcoholic NaOH. The mixture was stirred at room temperature for 1 h to afford (*E*)-5-(4-methyl­benzyl­idene)-1-phenyl-1,5,6,7-tetra­hydro-4*H*-ind­a­z­ol-4-ones as a precipitate, which was filtered, dried and recrystallized from ethanol: yield: 99%, m.p. 172–175°C.

## Refinement

Crystal data, data collection and structure refinement details are summarized in Table 2 ▸.

## Supplementary Material

Crystal structure: contains datablock(s) I. DOI: 10.1107/S2414314622002838/tk4075sup1.cif

Structure factors: contains datablock(s) I. DOI: 10.1107/S2414314622002838/tk4075Isup2.hkl

Supporting information file. DOI: 10.1107/S2414314622002838/tk4075Isup3.cml

CCDC reference: 2158365

Additional supporting information:  crystallographic information; 3D view; checkCIF report

## Figures and Tables

**Table 1:** Hydrogen-bond geometry (Å, °)

*D*—H⋯*A*	*D*—H	H⋯*A*	*D*⋯*A*	*D*—H⋯*A*
C6—H12⋯O1	0.93	2.43	2.806 (2)	104
C12—H4⋯O1^i^	0.93	2.52	3.312 (2)	143
C17—H5⋯O1^ii^	0.93	2.60	3.5081 (19)	164
C18—H8⋯O1^iii^	0.93	2.46	3.325 (2)	155

Symmetry codes: (i) 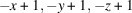 ; (ii) 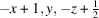 ; (iii) 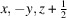 .

**Table 2:** Experimental details

Crystal data	
Chemical formula	C_21_H_18_N_2_O
*M*_r_	314.37
Crystal system, space group	Monoclinic, *C*2/*c*
Temperature (K)	293
*a*, *b*, *c* (Å)	30.3989 (15), 8.7177 (5), 14.0581 (7)
β (°)	115.367 (2)
*V* (Å^3^)	3366.3 (3)
*Z*	8
Radiation type	Mo *K*α
μ (mm^−1^)	0.08
Crystal size (mm)	0.20 × 0.20 × 0.18

Data collection	
Diffractometer	Bruker *SMART* APEXII CCD
Absorption correction	–
No. of measured, independent and observed [*I* > 2σ(*I*)] reflections	22457, 2948, 2557
*R*_int_	0.048
(sin θ/λ)_max_ (Å^−1^)	0.595

Refinement	
*R*[*F* ^2^ > 2σ(*F* ^2^)], *wR*(*F* ^2^), *S*	0.044, 0.126, 1.07
No. of reflections	2948
No. of parameters	219
H-atom treatment	H-atom parameters constrained
Δρ_max_, Δρ_min_ (e Å^−3^)	0.16, −0.18

Computer programs: *APEX2* and *SAINT* (Bruker, 2009 ▸), *SHELXT* (Sheldrick, 2015*a* ▸), *SHELXL* (Sheldrick, 2015*b* ▸), *ORTEP-3 for Windows* (Farrugia, 2012 ▸), *Mercury* (Macrae *et al.*, 2020 ▸) and *PLATON* (Spek, 2020 ▸).

## full crystallographic data

### Crystal data

**Table:**

C_21_H_18_N_2_O	*F*(000) = 1328
*M**_r_* = 314.37	*D*_x_ = 1.241 Mg m^−^^3^
Monoclinic, *C*2/*c*	Mo *K*α radiation, λ = 0.71073 Å
*a* = 30.3989 (15) Å	Cell parameters from 3243 reflections
*b* = 8.7177 (5) Å	θ = 28.7–1.8°
*c* = 14.0581 (7) Å	µ = 0.08 mm^−^^1^
β = 115.367 (2)°	*T* = 293 K
*V* = 3366.3 (3) Å^3^	Block, colourless
*Z* = 8	0.20 × 0.20 × 0.18 mm

### Data collection

**Table:**

Bruker SMART APEXII CCD diffractometer	2557 reflections with *I* > 2σ(*I*)
Radiation source: fine-focus sealed tube	*R*_int_ = 0.048
Graphite monochromator	θ_max_ = 25.0°, θ_min_ = 2.9°
ω and φ scans	*h* = −36→36
22457 measured reflections	*k* = −10→10
2948 independent reflections	*l* = −16→16

### Refinement

**Table:**

Refinement on *F*^2^	Secondary atom site location: difference Fourier map
Least-squares matrix: full	Hydrogen site location: inferred from neighbouring sites
*R*[*F*^2^ > 2σ(*F*^2^)] = 0.044	H-atom parameters constrained
*wR*(*F*^2^) = 0.126	*w* = 1/[σ^2^(*F*_o_^2^) + (0.0626*P*)^2^ + 1.8343*P*] where *P* = (*F*_o_^2^ + 2*F*_c_^2^)/3
*S* = 1.07	(Δ/σ)_max_ < 0.001
2948 reflections	Δρ_max_ = 0.16 e Å^−^^3^
219 parameters	Δρ_min_ = −0.17 e Å^−^^3^
0 restraints	Extinction correction: SHELXL, Fc^*^=kFc[1+0.001xFc^2^λ^3^/sin(2θ)]^-1/4^
Primary atom site location: structure-invariant direct methods	Extinction coefficient: 0.075 (5)

### Special details

**Table:**

Geometry. All esds (except the esd in the dihedral angle between two l.s. planes) are estimated using the full covariance matrix. The cell esds are taken into account individually in the estimation of esds in distances, angles and torsion angles; correlations between esds in cell parameters are only used when they are defined by crystal symmetry. An approximate (isotropic) treatment of cell esds is used for estimating esds involving l.s. planes.

### Fractional atomic coordinates and isotropic or equivalent isotropic displacement parameters (Å^2^)

**Table:**

	*x*	*y*	*z*	*U*_iso_*/*U*_eq_	
C1	0.67927 (8)	0.0162 (3)	1.06046 (15)	0.0789 (6)	
H1	0.6816	−0.0935	1.0667	0.118*	
H9	0.6614	0.0543	1.0977	0.118*	
H10	0.7114	0.0599	1.0900	0.118*	
C2	0.65323 (6)	0.0598 (2)	0.94602 (13)	0.0543 (4)	
C3	0.66930 (6)	0.1813 (2)	0.90557 (14)	0.0586 (5)	
H11	0.6970	0.2352	0.9496	0.070*	
C4	0.64503 (6)	0.2237 (2)	0.80118 (14)	0.0550 (4)	
H6	0.6572	0.3038	0.7758	0.066*	
C5	0.60276 (5)	0.14887 (18)	0.73320 (12)	0.0450 (4)	
C6	0.57687 (6)	0.20157 (19)	0.62334 (12)	0.0487 (4)	
H12	0.5965	0.2260	0.5899	0.058*	
C7	0.52919 (6)	0.21900 (18)	0.56538 (11)	0.0445 (4)	
C8	0.48996 (6)	0.1884 (2)	0.60232 (12)	0.0516 (4)	
H13	0.5052	0.1753	0.6781	0.062*	
H14	0.4736	0.0931	0.5713	0.062*	
C9	0.45177 (5)	0.3169 (2)	0.57388 (11)	0.0462 (4)	
H15	0.4246	0.2838	0.5876	0.055*	
H16	0.4660	0.4074	0.6160	0.055*	
C10	0.43483 (5)	0.35272 (17)	0.45978 (11)	0.0416 (4)	
C11	0.34983 (5)	0.45572 (19)	0.40541 (12)	0.0487 (4)	
C12	0.35632 (6)	0.5344 (2)	0.49555 (13)	0.0541 (4)	
H4	0.3875	0.5536	0.5471	0.065*	
C13	0.31616 (7)	0.5847 (2)	0.50901 (16)	0.0657 (5)	
H3	0.3205	0.6369	0.5701	0.079*	
C14	0.27001 (7)	0.5582 (3)	0.43271 (17)	0.0761 (6)	
H2	0.2431	0.5928	0.4417	0.091*	
C15	0.51236 (6)	0.27563 (18)	0.45440 (11)	0.0449 (4)	
C16	0.46258 (6)	0.32941 (18)	0.40502 (11)	0.0440 (4)	
C17	0.43202 (6)	0.3710 (2)	0.30018 (12)	0.0518 (4)	
H5	0.4413	0.3674	0.2453	0.062*	
C18	0.61226 (6)	−0.01863 (19)	0.87812 (13)	0.0545 (4)	
H8	0.6013	−0.1025	0.9029	0.065*	
C19	0.58722 (6)	0.02523 (18)	0.77400 (13)	0.0509 (4)	
H7	0.5595	−0.0287	0.7304	0.061*	
C20	0.30333 (6)	0.4275 (3)	0.32794 (14)	0.0679 (5)	
H18	0.2989	0.3743	0.2671	0.081*	
C21	0.26374 (7)	0.4799 (3)	0.34264 (17)	0.0825 (7)	
H17	0.2324	0.4620	0.2910	0.099*	
N1	0.39101 (4)	0.40554 (15)	0.38972 (9)	0.0463 (3)	
N2	0.38894 (5)	0.41551 (17)	0.28944 (10)	0.0550 (4)	
O1	0.53899 (4)	0.27719 (16)	0.40893 (9)	0.0624 (4)	

### Atomic displacement parameters (Å^2^)

**Table:**

	*U*^11^	*U*^22^	*U*^33^	*U*^12^	*U*^13^	*U*^23^
C1	0.0871 (14)	0.0832 (14)	0.0504 (11)	0.0112 (11)	0.0142 (10)	0.0108 (10)
C2	0.0548 (9)	0.0552 (10)	0.0472 (9)	0.0125 (7)	0.0163 (7)	0.0056 (7)
C3	0.0443 (8)	0.0577 (10)	0.0587 (10)	0.0017 (7)	0.0079 (7)	0.0034 (8)
C4	0.0442 (8)	0.0548 (10)	0.0624 (10)	0.0039 (7)	0.0192 (7)	0.0142 (8)
C5	0.0442 (8)	0.0470 (9)	0.0442 (8)	0.0097 (6)	0.0194 (6)	0.0042 (6)
C6	0.0527 (9)	0.0532 (9)	0.0453 (8)	0.0075 (7)	0.0259 (7)	0.0052 (7)
C7	0.0510 (8)	0.0484 (8)	0.0371 (7)	0.0075 (6)	0.0218 (6)	0.0038 (6)
C8	0.0522 (9)	0.0645 (10)	0.0409 (8)	0.0091 (7)	0.0226 (7)	0.0154 (7)
C9	0.0449 (8)	0.0614 (9)	0.0339 (7)	0.0044 (7)	0.0183 (6)	0.0071 (6)
C10	0.0443 (7)	0.0444 (8)	0.0329 (7)	−0.0007 (6)	0.0134 (6)	0.0012 (6)
C11	0.0432 (8)	0.0558 (9)	0.0430 (8)	0.0023 (7)	0.0146 (6)	0.0117 (7)
C12	0.0455 (8)	0.0617 (10)	0.0509 (9)	0.0024 (7)	0.0168 (7)	0.0010 (8)
C13	0.0602 (10)	0.0758 (13)	0.0659 (11)	0.0090 (9)	0.0317 (9)	0.0046 (9)
C14	0.0502 (10)	0.1054 (17)	0.0754 (14)	0.0144 (10)	0.0296 (10)	0.0224 (12)
C15	0.0550 (9)	0.0479 (9)	0.0366 (7)	0.0026 (7)	0.0244 (7)	−0.0001 (6)
C16	0.0547 (8)	0.0465 (8)	0.0313 (7)	0.0011 (6)	0.0191 (6)	0.0003 (6)
C17	0.0654 (10)	0.0582 (10)	0.0324 (8)	0.0072 (8)	0.0214 (7)	0.0027 (7)
C18	0.0589 (9)	0.0478 (9)	0.0540 (9)	0.0043 (7)	0.0217 (8)	0.0100 (7)
C19	0.0505 (9)	0.0457 (9)	0.0493 (9)	0.0013 (7)	0.0145 (7)	0.0004 (7)
C20	0.0513 (10)	0.0972 (15)	0.0440 (9)	−0.0042 (9)	0.0097 (7)	0.0053 (9)
C21	0.0429 (10)	0.128 (2)	0.0619 (12)	−0.0002 (11)	0.0088 (8)	0.0193 (13)
N1	0.0480 (7)	0.0545 (8)	0.0324 (6)	0.0029 (6)	0.0134 (5)	0.0035 (5)
N2	0.0639 (9)	0.0638 (9)	0.0313 (7)	0.0062 (7)	0.0146 (6)	0.0044 (6)
O1	0.0667 (8)	0.0851 (9)	0.0476 (7)	0.0143 (6)	0.0361 (6)	0.0098 (6)

### Geometric parameters (Å, º)

**Table:**

C1—C2	1.506 (2)	C10—C16	1.379 (2)
C1—H1	0.9600	C11—C12	1.379 (2)
C1—H9	0.9600	C11—C20	1.388 (2)
C1—H10	0.9600	C11—N1	1.430 (2)
C2—C18	1.382 (2)	C12—C13	1.384 (2)
C2—C3	1.386 (3)	C12—H4	0.9300
C3—C4	1.381 (2)	C13—C14	1.372 (3)
C3—H11	0.9300	C13—H3	0.9300
C4—C5	1.392 (2)	C14—C21	1.378 (3)
C4—H6	0.9300	C14—H2	0.9300
C5—C19	1.395 (2)	C15—O1	1.2279 (18)
C5—C6	1.474 (2)	C15—C16	1.446 (2)
C6—C7	1.333 (2)	C16—C17	1.412 (2)
C6—H12	0.9300	C17—N2	1.311 (2)
C7—C15	1.502 (2)	C17—H5	0.9300
C7—C8	1.514 (2)	C18—C19	1.383 (2)
C8—C9	1.538 (2)	C18—H8	0.9300
C8—H13	0.9700	C19—H7	0.9300
C8—H14	0.9700	C20—C21	1.383 (3)
C9—C10	1.4925 (19)	C20—H18	0.9300
C9—H15	0.9700	C21—H17	0.9300
C9—H16	0.9700	N1—N2	1.3867 (17)
C10—N1	1.3535 (18)		
			
C2—C1—H1	109.5	C16—C10—C9	123.85 (13)
C2—C1—H9	109.5	C12—C11—C20	120.41 (16)
H1—C1—H9	109.5	C12—C11—N1	120.27 (13)
C2—C1—H10	109.5	C20—C11—N1	119.30 (15)
H1—C1—H10	109.5	C11—C12—C13	119.73 (16)
H9—C1—H10	109.5	C11—C12—H4	120.1
C18—C2—C3	117.79 (15)	C13—C12—H4	120.1
C18—C2—C1	121.21 (17)	C14—C13—C12	120.38 (19)
C3—C2—C1	120.99 (17)	C14—C13—H3	119.8
C4—C3—C2	121.26 (16)	C12—C13—H3	119.8
C4—C3—H11	119.4	C13—C14—C21	119.64 (18)
C2—C3—H11	119.4	C13—C14—H2	120.2
C3—C4—C5	121.32 (16)	C21—C14—H2	120.2
C3—C4—H6	119.3	O1—C15—C16	122.29 (13)
C5—C4—H6	119.3	O1—C15—C7	122.50 (14)
C4—C5—C19	117.09 (14)	C16—C15—C7	115.20 (12)
C4—C5—C6	119.64 (14)	C10—C16—C17	104.99 (13)
C19—C5—C6	123.27 (14)	C10—C16—C15	122.98 (13)
C7—C6—C5	128.94 (14)	C17—C16—C15	132.02 (14)
C7—C6—H12	115.5	N2—C17—C16	111.97 (14)
C5—C6—H12	115.5	N2—C17—H5	124.0
C6—C7—C15	118.02 (14)	C16—C17—H5	124.0
C6—C7—C8	125.46 (13)	C2—C18—C19	121.22 (16)
C15—C7—C8	116.51 (13)	C2—C18—H8	119.4
C7—C8—C9	113.58 (13)	C19—C18—H8	119.4
C7—C8—H13	108.8	C18—C19—C5	121.25 (15)
C9—C8—H13	108.8	C18—C19—H7	119.4
C7—C8—H14	108.8	C5—C19—H7	119.4
C9—C8—H14	108.8	C21—C20—C11	118.89 (19)
H13—C8—H14	107.7	C21—C20—H18	120.6
C10—C9—C8	107.83 (12)	C11—C20—H18	120.6
C10—C9—H15	110.1	C14—C21—C20	120.94 (18)
C8—C9—H15	110.1	C14—C21—H17	119.5
C10—C9—H16	110.1	C20—C21—H17	119.5
C8—C9—H16	110.1	C10—N1—N2	111.28 (12)
H15—C9—H16	108.5	C10—N1—C11	130.21 (12)
N1—C10—C16	106.89 (12)	N2—N1—C11	118.44 (12)
N1—C10—C9	129.20 (13)	C17—N2—N1	104.86 (12)
			
C18—C2—C3—C4	0.6 (3)	O1—C15—C16—C10	−168.78 (15)
C1—C2—C3—C4	−178.75 (18)	C7—C15—C16—C10	10.8 (2)
C2—C3—C4—C5	1.8 (3)	O1—C15—C16—C17	10.0 (3)
C3—C4—C5—C19	−2.7 (2)	C7—C15—C16—C17	−170.40 (16)
C3—C4—C5—C6	177.62 (15)	C10—C16—C17—N2	−0.38 (19)
C4—C5—C6—C7	−137.43 (18)	C15—C16—C17—N2	−179.36 (16)
C19—C5—C6—C7	42.9 (3)	C3—C2—C18—C19	−1.9 (3)
C5—C6—C7—C15	179.80 (15)	C1—C2—C18—C19	177.42 (17)
C5—C6—C7—C8	0.7 (3)	C2—C18—C19—C5	0.9 (3)
C6—C7—C8—C9	133.31 (17)	C4—C5—C19—C18	1.3 (2)
C15—C7—C8—C9	−45.8 (2)	C6—C5—C19—C18	−178.96 (15)
C7—C8—C9—C10	49.38 (18)	C12—C11—C20—C21	0.1 (3)
C8—C9—C10—N1	150.39 (16)	N1—C11—C20—C21	−178.39 (18)
C8—C9—C10—C16	−26.6 (2)	C13—C14—C21—C20	0.0 (4)
C20—C11—C12—C13	0.4 (3)	C11—C20—C21—C14	−0.3 (3)
N1—C11—C12—C13	178.88 (16)	C16—C10—N1—N2	0.89 (17)
C11—C12—C13—C14	−0.7 (3)	C9—C10—N1—N2	−176.49 (15)
C12—C13—C14—C21	0.5 (3)	C16—C10—N1—C11	−176.18 (15)
C6—C7—C15—O1	14.8 (2)	C9—C10—N1—C11	6.4 (3)
C8—C7—C15—O1	−166.01 (16)	C12—C11—N1—C10	36.0 (2)
C6—C7—C15—C16	−164.78 (15)	C20—C11—N1—C10	−145.48 (17)
C8—C7—C15—C16	14.4 (2)	C12—C11—N1—N2	−140.90 (16)
N1—C10—C16—C17	−0.32 (17)	C20—C11—N1—N2	37.6 (2)
C9—C10—C16—C17	177.24 (15)	C16—C17—N2—N1	0.89 (19)
N1—C10—C16—C15	178.78 (14)	C10—N1—N2—C17	−1.10 (18)
C9—C10—C16—C15	−3.7 (2)	C11—N1—N2—C17	176.35 (14)

### Hydrogen-bond geometry (Å, º)

**Table:**

*D*—H···*A*	*D*—H	H···*A*	*D*···*A*	*D*—H···*A*
C6—H12···O1	0.93	2.43	2.806 (2)	104
C12—H4···O1^i^	0.93	2.52	3.312 (2)	143
C17—H5···O1^ii^	0.93	2.60	3.5081 (19)	164
C18—H8···O1^iii^	0.93	2.46	3.325 (2)	155

Symmetry codes: (i) −*x*+1, −*y*+1, −*z*+1; (ii) −*x*+1, *y*, −*z*+1/2; (iii) *x*, −*y*, *z*+1/2.
